# Seroprevalence of dengue, Zika, chikungunya and Ross River viruses across the Solomon Islands

**DOI:** 10.1371/journal.pntd.0009848

**Published:** 2022-02-10

**Authors:** Tanya L. Russell, Paul F. Horwood, Humpress Harrington, Allan Apairamo, Nathan J. Kama, Albino Bobogare, David MacLaren, Thomas R. Burkot

**Affiliations:** 1 Australian Institute of Tropical Health and Medicine, James Cook University, Cairns, Australia; 2 College of Public Health, Medical and Veterinary Sciences, James Cook University, Townsville, Australia; 3 College of Medicine and Dentistry, James Cook University, Cairns, Australia; 4 Atoifi College of Nursing, Atoifi Adventist Hospital, Atoifi, Malaita, Solomon Islands; 5 National Vector Borne Disease Control Program, Ministry of Health and Medical Services, Honiara, Solomon Islands; University of Malaya Faculty of Medicine, MALAYSIA

## Abstract

Across the Pacific, and including in the Solomon Islands, outbreaks of arboviruses such as dengue, chikungunya, and Zika are increasing in frequency, scale and impact. Outbreaks of mosquito-borne disease have the potential to overwhelm the health systems of small island nations. This study mapped the seroprevalence of dengue, Zika, chikungunya and Ross River viruses in 5 study sites in the Solomon Islands. Serum samples from 1,021 participants were analysed by ELISA. Overall, 56% of participants were flavivirus-seropositive for dengue (28%), Zika (1%) or both flaviviruses (27%); and 53% of participants were alphavirus-seropositive for chikungunya (3%), Ross River virus (31%) or both alphaviruses (18%). Seroprevalence for both flaviviruses and alphaviruses varied by village and age of the participant. The most prevalent arboviruses in the Solomon Islands were dengue and Ross River virus. The high seroprevalence of dengue suggests that herd immunity may be a driver of dengue outbreak dynamics in the Solomon Islands. Despite being undetected prior to this survey, serology results suggest that Ross River virus transmission is endemic. There is a real need to increase the diagnostic capacities for each of the arboviruses to support effective case management and to provide timely information to inform vector control efforts and other outbreak mitigation interventions.

## Author summary

The occurrence of arboviruses is increasing and causing significant impacts on human health. This is of high concern in small Pacific island nations where fragile health systems are regularly overwhelmed by disease outbreaks. To effectively prevent and control disease transmission there is a need to understand which viruses have been in circulation. Therefore, we conducted a cross-sectional survey of residents from 5 study sites distributed across the Solomon Islands. The serum samples were tested for antibodies that indicate prior infection for four arboviruses. We found evidence that the residents of the Solomon Islands have been exposed to substantial transmission of dengue and Ross River viruses, with lower levels of Zika and chikungunya transmission. Two large dengue outbreaks have been recently experienced and the outbreak pattern suggests that natural herd immunity may still be a driver of dengue outbreak dynamics in the Solomon Islands. Regarding Ross River virus, transmission is endemic despite being undetected prior to this survey. There is a real need to increase the capacity to accurately diagnose each of these arboviruses to support effective case management and to provide timely information to inform vector control efforts.

## Background

Globally, mosquito-borne diseases are increasing in geographic distribution and incidence [1]. Dengue incidence increased 30-fold globally over the past 50 years, with transmission spreading into many new countries [2,3]. The drivers of this epidemiological trend includes anthropogenic factors such as increased travel, land-use change and climate change [1,4,5]. The threat of increasing mosquito borne diseases is of high concern to the small Pacific island countries’ fragile health systems where outbreaks of arboviruses, including dengue (DENV), Zika (ZIKV), chikungunya (CHIKV) and Ross River (RRV) viruses, are increasing in frequency, scale and impact [6]. Patients infected with these arboviruses present with overlapping symptoms, making symptomatic diagnosis unreliable. Asymptomatic and mild clinical forms of DENV, ZIKV, CHIKV and RRV may account for a large proportion of all infections. When present, initial symptoms include fever, headache, myalgia, arthralgia, maculopapular rash and lymphadenopathies [7,8].

The Pacific region has the highest diversity of DENV, CHIKV and ZIKV vectors in the world. Many of these species are important in limited geographies, or play roles as secondary vectors because of their catholic feeding behaviour. DENV, ZIKV and CHIKV are predominantly transmitted by *Ae*. *aegypti*, but can also be transmitted by *Ae*. *albopictus* or other *Aedes* species (predominately from the *Stegomyia* subgenus). The distributions of *Ae*. *aegypti* and *Ae*. *albopictus* have been expanding globally, and across the region [9]. *Aedes albopictus* was detected in northern Papua New Guinea in the early 1970s [10] and then in Solomon Islands in 1979 [11]. There were no further records of *Ae*. *aegypti* in Papua New Guinea or Solomon Islands for 35 years, until being reported again in 2013 [12,13]. Ross River virus is transmitted by a wide range of both *Aedes* and *Culex* species [14], which are ubiquitous across the Pacific.

In the five-year period ending June 2019, 36,270 cases of dengue-like illnesses were reported from 14 Pacific Island Countries and Territories (PICTs) to the Pacific Syndromic Surveillance System. Most cases across the Pacific were vectored by *Ae*. *aegypti*, however in the Solomon Islands, large DENV outbreaks were vectored by both *Ae*. *aegypti* and/or *Ae*. *albopictus* in both 2013 and 2016–17 [15–17].

In the Pacific, chikungunya was reported from Papua New Guinea in 1969 [18], and was then largely absent from the PICTs until an outbreak in New Caledonia in 2011 [19,20]. Chikungunya outbreaks or cases have since been reported from Papua New Guinea, Federated States of Micronesia, Tonga, American Samoa, Samoa, Tokelau [6], French Polynesia [21] and Solomon Islands [17,22]. *Ae*. *aegypti* and *Ae*. *albopictus* are the principal vectors [19].

Zika virus was absent from the PICTs until the first major ZIKV outbreak occurred on Yap Island, Federated States of Micronesia, in 2007 [23]. This was followed by a large outbreak in French Polynesia in 2013–14 associated with a rise in Guillain-Barré syndrome cases [24,25]. Transmission of ZIKV has now been recorded from at least 20 PICTs including New Caledonia, Cook Islands, Vanuatu, Fiji, Tonga, Papua New Guinea and the Solomon Islands [26–28].

Ross River virus is considered to be endemic to Australia and Papua New Guinea, and historically marsupials were thought to be the primary zoonotic reservoir hosts [29]. This dogma lead to the belief that RRV could not circulate in the Pacific. However, recent modelling efforts have clarified that interactions between hosts and vectors largely underpin the importance of host species, and that placental mammals, including humans, and birds play important roles in RRV transmission cycles [30,31]. RRV transmission has actually been detected in Fiji, Cook Islands, American Samoa, New Caledonia, Wallis & Fatuna, French Polynesia and Vanuatu [32–39] with placental mammals (including horses, pigs and rats) believed to be zoonotic reservoirs [35–40]. Low-level RRV circulation is likely to be occurring undetected in many Pacific countries [39].

In the Solomon Islands, little is known about the geographic distribution of DENV, ZIKV, CHIKV and RRV. These mosquito-borne diseases have no treatment and therefore prevention and control relies on reducing vector populations or preventing human exposure to mosquito bites. As such, understanding transmission dynamics and geographical distribution is essential for stratifying areas to target resources to effectively implement proactive control as well as to respond rapidly to outbreaks [41,42]. As such, this study mapped the exposure prevalence to DENV, ZIKV, CHIKV and RRV in five provinces in the Solomon Islands. The primary aim of the study was to investigate the variability in arbovirus transmission across the country.

## Methods

### Ethics statement

Community meetings were held with all village residents prior to the survey, where the aims, the possible risks and potential benefits of the study were explained in Solomon Islands Pidgin. Participation was voluntary with written informed consent obtained prior to enrolment from each adult participant >18 years. For children between 13 and 18 years, signed consent was obtained from both the minor and a parent or guardian; for children between 5 and 13 years, signed consent was obtained from a parent or guardian. The results of the CareStart RDTs for malaria were immediately provided to the participants.

Ethical approvals for the study were obtained from the National Health Research & Ethics Committee, Solomon Islands (HRE066/17) and the James Cook University Human Research Ethics Committee, Australia (H7107). The field sampling and subsequent analyses was performed in accordance with relevant guidelines and regulations of these research boards as stipulated in the approvals.

### Study sites and period

A cross-sectional study was conducted in the Solomon Islands (-8.0° S, 157.0° E). The Solomon Islands is hot and wet with an annual rainfall of 2,005 mm (mean for 1999–2017 at Henderson Airport, Guadalcanal Island). The mean daily coastal temperature ranges between 24°C and 30°C with a mean of 26°C.

**Fig 1:**
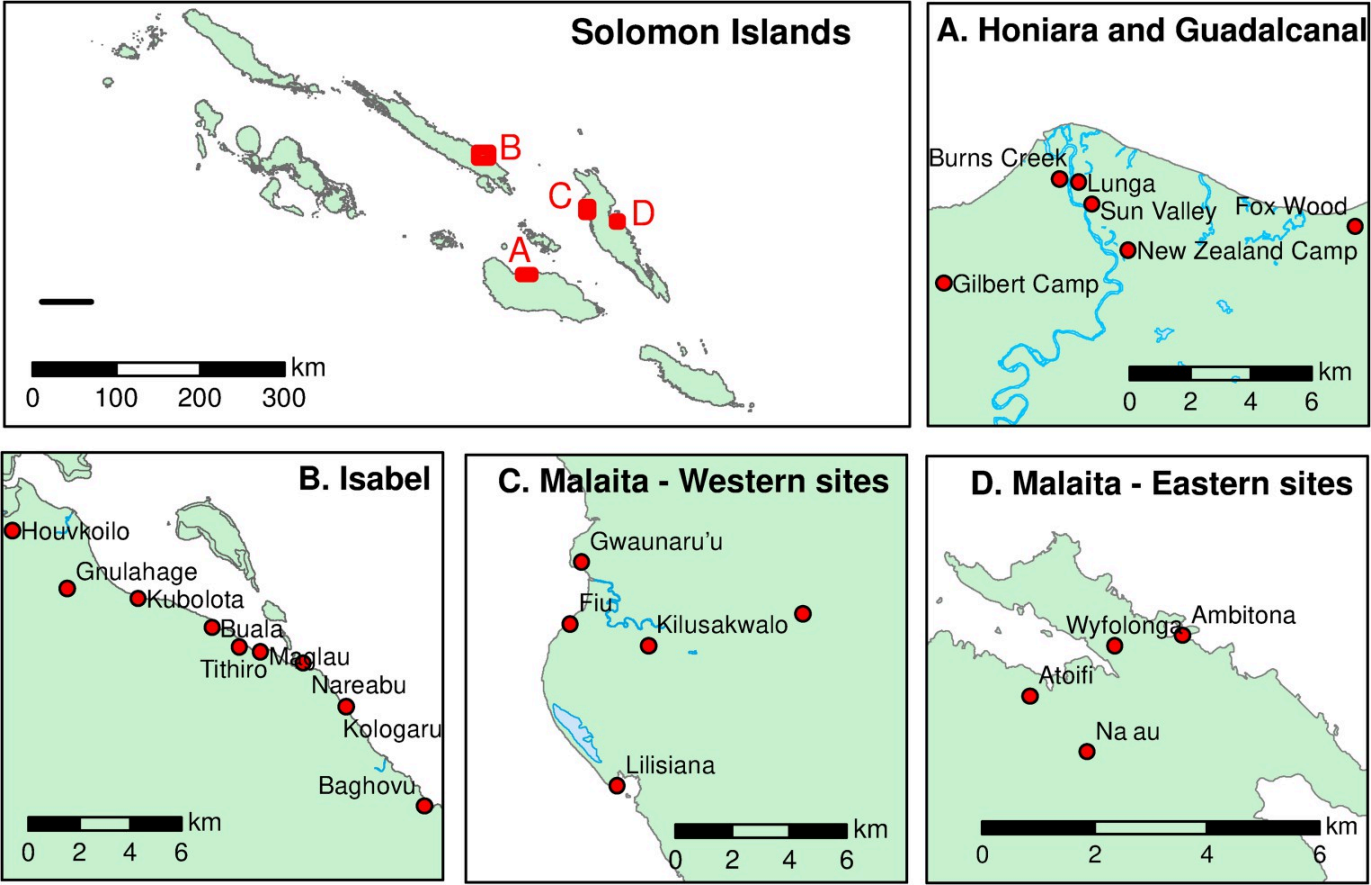
**Map of the Solomon Islands (-8.0° S, 157.0° E) showing villages in the study sites of: A) Guadalcanal, B) Isabel, C) West Malaita, and D) East Malaita.** The base map was obtained from http://diva-gis.org/data.

The first stage of the study design involved selecting the study sites, and villages within each site. The five study sites were selected in consultation with the Ministry of Health and Medical Services, Solomon Islands. Historical syndromic surveillance data for dengue like illness indicated that the majority of past DENV transmission was focused in the capital city and northern Guadalcanal; however, the level of transmission in the remote and regional areas was unclear. As such, the study sites were strategically selected to include these differing regions: Honiara, North Guadalcanal, Isabel, East Malaita and West Malaita. Within each study site, multiple villages were selected. Inclusion criteria required villages to have a minimum resident population of 200, and to be accessible by sea or road. The sites encompassed 23 suburbs and villages (Fig 1), hereforth termed villages. Honiara, Guadalcanal, Isabel and West Malaita were surveyed in April 2018, and East Malaita was surveyed in November 2018.

### Field procedures

Within each village, all residents over the age of 5 years, were invited to participate in the study. Village residents were contacted and recruited via village meetings and with the support of village leaders. The only exclusion criteria was the resident’s unwillingness to participate in the study. Residents were equally encouraged to participate across age categories and genders, with a target of 100 residents per village. After the samples were collected, a subset was forwarded for arbovirus analysis. The subset was identified using blinded random selection that was stratified to select an equal number of participants from each village.

After enrolment, demographic information and data on possible risk and protective factors associated with mosquito-borne diseases of participants were collected. Data collected included: (1) name, age, sex, household number, (2) domestic and international travel history, (3) fever history and (4) access/use of mosquito protection measures (use of insecticide treated bednets [ITNs], house with window-screens or recently treated by indoor residual spraying of insecticides, topical repellents and spatial repellents [mosquito coils]). The typanic temperature of participants was measured (Welch Allyn Braun ThermoScan Pro 6000) and any febrile individuals (temperatures >38°C) were immediately referred to the nearest health facility.

Each participant provided a ≤10 ml blood sample by venepuncture using vacutainers (BD K2EDTA plasma vacutainers), drawn by a Ministry of Health and Medical Services nurse. Five μl of each blood sample was immediately tested for malaria using the AccessBio CareStart rapid diagnostic test (RDT) (G0161) according to the manufacturer’s instructions. Concurrently, 3 × 50 μl blood spots were placed onto cellulose chromatography papers (2 × 7 cm; Whatman Grade 3MM) and dried under ambient conditions. The malaria data has been analysed elsewhere [43].

Serum was separated by centrifugation at 1,500 g for 10 minutes. Serum and clots were initially stored at 4°C, then frozen at −20°C within 4 days, until shipped internationally on dry ice and subsequently stored at −80°C until analysed. A unique code was assigned to each participant and their associated samples.

### ELISA detection of arbovirus antibodies

Serum samples were tested by enzyme-linked immunosorbent assays (ELISAs) for IgG antibodies against DENV, ZIKV, CHIKV and RRV using the Dengue IgG Indirect ELISA (Panbio Ltd, Brisbane, Australia), Anti-Zika Virus ELISA IgG (Euroimmun, Lübeck, Germany), Anti-Chikungunya Virus ELISA IgG (Euroimmun, Lübeck, Germany) and Ross River virus IgG ELISA (Panbio Ltd, Brisbane, Australia). Optical density (OD) values were measured for each sample using dual wavelength readings at 450 nm/650 nm with a FLUOstar Optima microplate reader (BMG Labtech, Offenburg, Germany) using Softmax Pro v6.5.1 software (Molecular Devices, Sunnyvale, CA, USA). For the Panbio assays (DENV and RRV) and the Euroimmun assays (ZIKV and CHIKV), signal-to-cutoff ratios were calculated following the manufacturer’s intructions. Samples generating values within the equivocal range were regarded as negative. The serological tests were conducted as recommended by the manufacturer’s with the inclusion of quality control measures (positive/negative controls and calibration samples) included with each plate to assess the validity of results.

### Statistical analysis

Models were fitted to examine the influence of explanatory factors for evidence of exposure to arboviruses as the binary dependent variable (i.e., negative or positive). Arbovirus seropositivity was analysed separately for flaviviruses (DENV and ZIKV) and alphaviruses (CHIKV and RRV). Individuals seropositive for both viruses within the flavi- or alphavirus families were coded as either flavi- or alphavirus antibody positive. Individuals seropositive for only one virus in a family were recorded as having antibodies to that virus.

The correlation between domestic and international travel history and village was analysed using chi-squared contingency tables (*chisq*.*test*). The strength of evidence for study site and village influence on seropositivity was compared in competing models constructed as a generalised linear model (GLM; package *MASS*) for site, compared with a generalised linear mixed model (GLMM; package *lme4*) for village with site as a random factor. The influence of explanatory variables for village, sex, temperature, age and bednet use were investigated using quantitative step-forward multi-model inference (MMI) selection procedures. Travel history was excluded from the model selection because it was strongly correlated with village. Model selection was based on ranking the value of the Akaike’s Information Criterion (AIC). The relative strength of evidence for each model within the set of alternatives was assessed using Akaike weights (*w*AIC) where the *w*AIC for each model is interpreted as the probability for the most likely model, with support varying from 0 (no support) to 1 (total support) [44–46]. The most parsimonious model from the final set of nested models was compared with the likelihood ratio test and compared with the *Χ*^2^ distribution [47,48]. Within the flavi- or alphaviruses, the difference in the proportion of solely seropositive residents for each arbovirus was examined with a 2-sample chi-squared test (*prop*.*test*). Analyses were performed using the R package (v3.5.1).

## Results

### Study population

A total of 2,393 individuals (215 from Honiara, 221 from Guadalcanal, 392 from Western Malaita, 416 from Eastern Malaita, and 996 from Isabel) participated in the study. Participants had a median age of 29 years, with 63% female (Table 1). The average tympanic temperature of participants was 37.1°C. A temperature exceeding 38°C was recorded in 40 people (1.7%). The maximum temperature recorded was 40.6°C.

**Table 1:** Study population summary characteristics.

Characteristic	Summary
Survey dates	Apr–Nov 2018
Number of participants	2,393
Age–Range	5–86 years
Age–Median	29 years
Percentage female	63% (n = 1,516)
Percent Flavivirus positive	56% (n = 569/1,021)
Honiara	82% (n = 76/92)
Guadalcanal	87% (n = 156/179)
East Malaita	46% (n = 73/158)
West Malaita	74% (n = 137/184)
Isabel	31% (n = 127/408)
Percent Alphavirus positive	53% (n = 537/1,021)
Honiara	60% (n = 56/92)
Guadalcanal	78% (n = 139/179)
East Malaita	47% (n = 75/158)
West Malaita	66% (n = 121/184)
Isabel	36% (n = 146/408)

### Travel history

Domestic travel within the two weeks prior to the survey was undertaken by 4.9% of participants and was significantly related to study sites (χ^2^ = 45.44, df = 4, p <0.0001; Fig A in S1 Text). Most frequent domestic travel was reported from participants in Honiara and East Malaita both with 9.3% (n = 20/215 and 39/417, respectively), compared to Isabel and West Malaita both with 2.5% (n = 25/971 and 10/392, respectively). International travel, at any time within their life, was undertaken by 2.9% of participants and was significantly related to study site (χ^2^ = 43.33, df = 4, p <0.0001; Fig B in S1 Text). International travel was reported most frequently by East Malaita residents (7%, n = 30/417) followed by Guadalcanal (5.8%, n = 22/374) and Honiara (4%, n = 9/205).

### Arbovirus serology

Serum samples from 1,021 participants were analysed by ELISA for antibodies to DENV, ZIKV, CHIKV and RRV. Overall, 569 participants were flavivirus-seropositive for DENV (n = 282), ZIKV (n = 11) or both flaviviruses (n = 276); 537 participants were alphavirus-seropositive for CHIKV (n = 31), RRV (n = 321) or both alphaviruses (n = 185) (Table 1).

**Fig 2:**
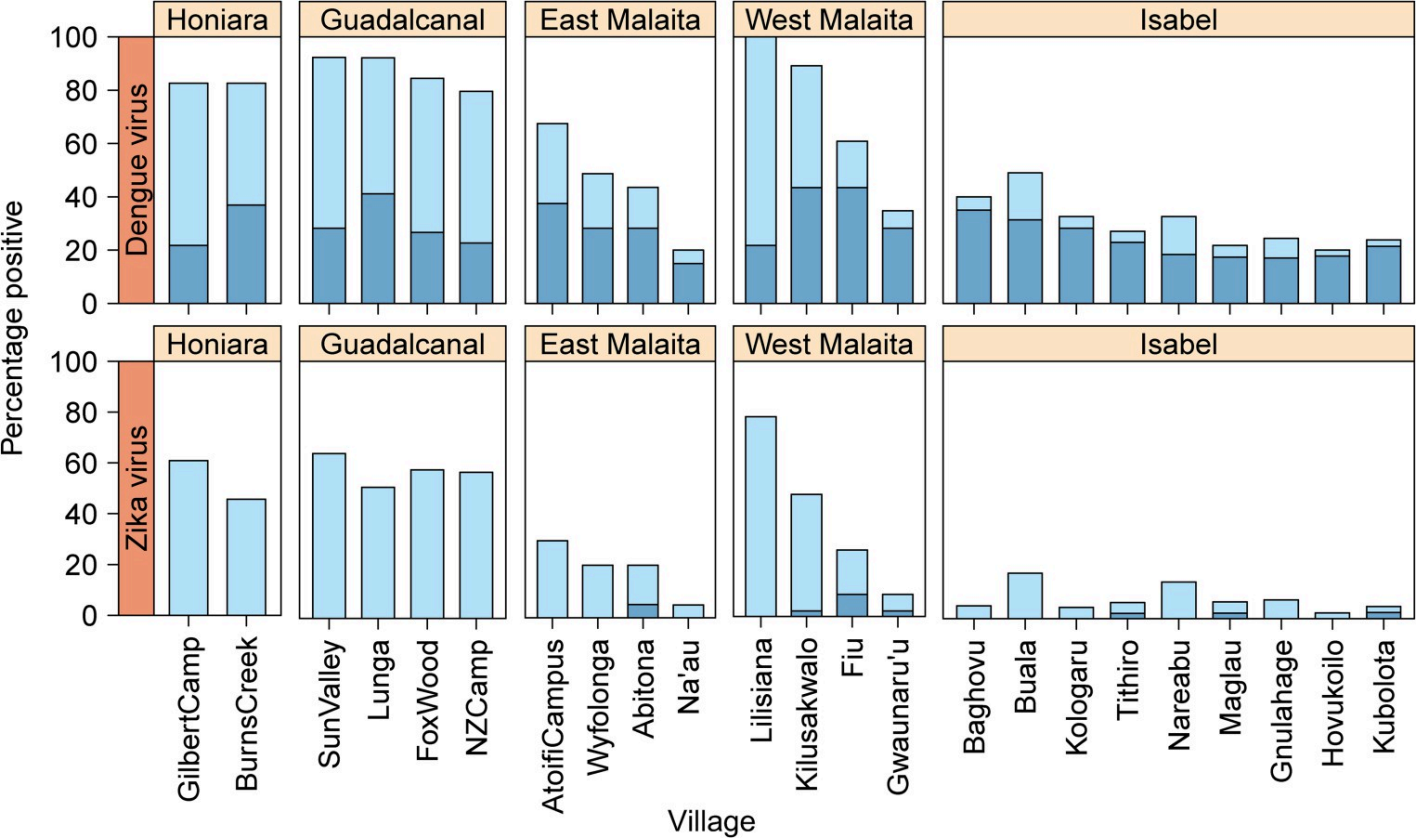
The seroprevalence of flaviviruses across sub-areas in the Solomon Islands. The darker blue colour represents samples that were flavivirus-positive for dengue or Zika alone. The lighter blue colour represents samples that were positive for both dengue and Zika.

**Fig 3:**
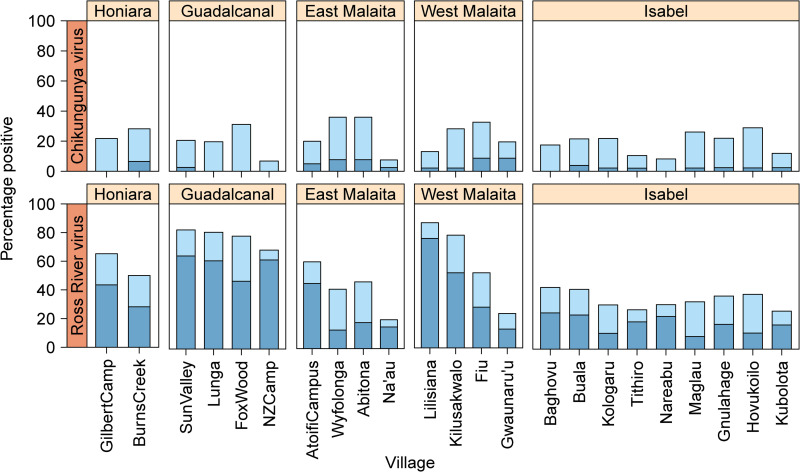
The seroprevalence of alphaviruses across villages in the Solomon Islands. The darker blue colour represents samples that were alphavirus-positive for chikungunya or Ross River virus alone. The lighter blue colour represents samples that were positive for both chikungunya and Ross River virus.

**Fig 4:**
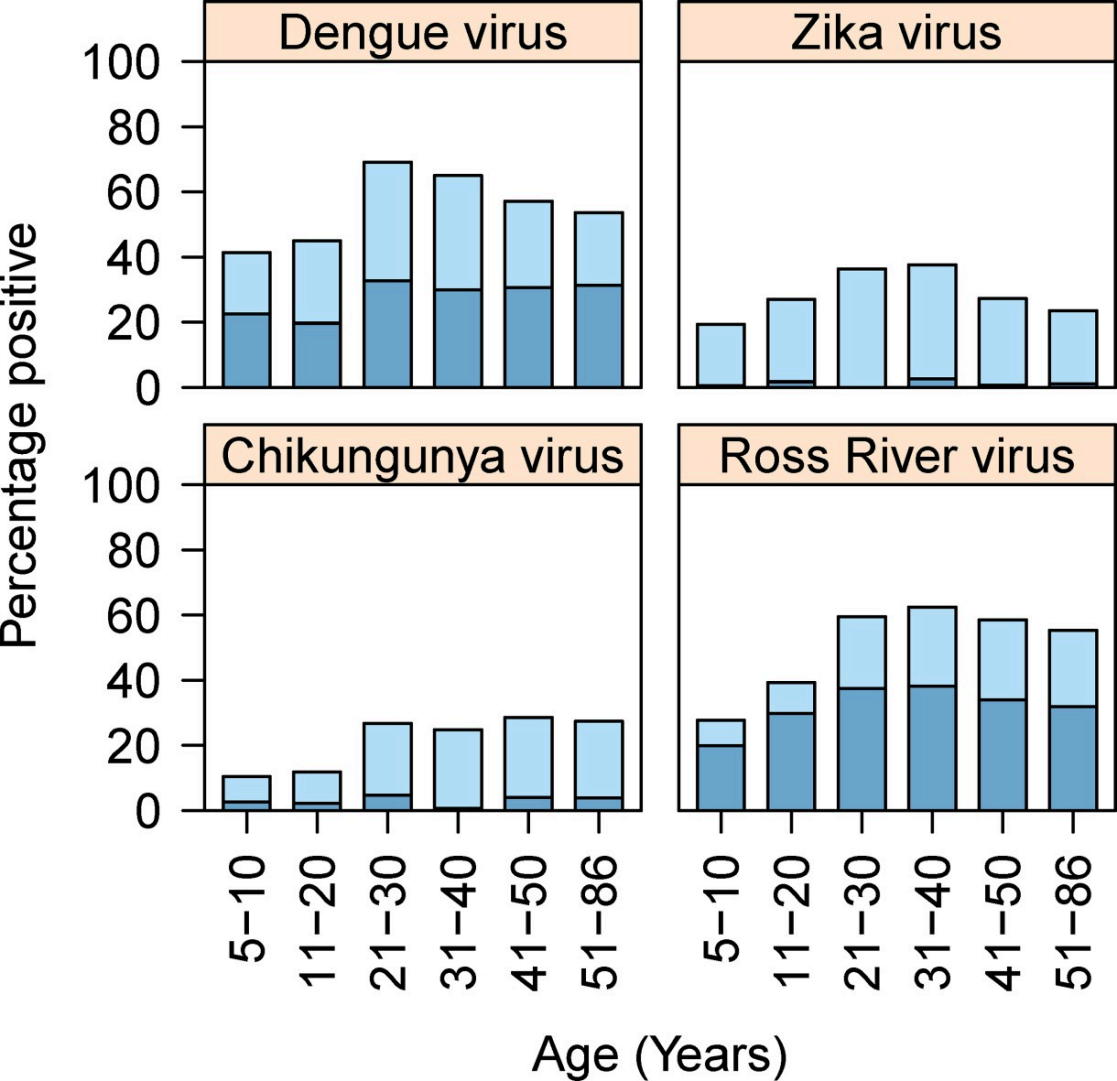
The seroprevalence of flaviviruses and alphavirus across different age groups in the Solomon Islands. The darker blue colour represents samples that were positive for that particular virus alone. The lighter blue colour represents samples that were positive for both of the flaviviruses (dengue and Zika) or both of the alphaviruses (chikungunya and Ross River virus).

Very similar results were recorded for both the flavivirus and alphavirus GLMs. For both, the base GLM model was most substantially improved by adding village (100% *w*AIC support, Figs 2 and 3). Sequentially both models were improved by adding age (99% *w*AIC support, Fig 4). These explanatory variables of village and age were significant (log-likelihood ratio test) and were included in the most parsimonious model (Table 2). None of the other remaining candidate factors were able to further improve model fit (Table 3).

**Table 2:** Final set of nested models evaluated to determine which best predicted the seroprevalence of flavi- and alphaviruses.

Model	df	AIC	ΔAIC	*w*AIC	χ^2^	*p* value
**Flavivirus**						
Village	24	1100.53	24.33	<0.0001		
Village + Age	25	1076.20	0.00	0.9987	26.33	<0.0001*
**Alphavirus**						
Village	24	1282.47	61.16	<0.0001		
Village + Age	25	1221.31	0	1.0000	63.15	<0.0001*

Model comparison was made on the basis ΔAIC, *w*AIC and goodness-of-fit using maximum likelihood estimation. The full list of explanatory variables included village, sex, temperature, age and bednet use.

**Table 3:** The number and percentage of participants that were positive flaviviruses or alphaviruses summarised by the various explanatory variables.

		Flavivirus		Alphavirus	
Parameter	Total	n	%	n	%
**Sex**					
Female	649	362	55.8%	344	53.0%
Male	372	207	55.6%	179	51.9%
**Fever**					
Yes	20	8	40.0%	6	30.0%
No	1001	561	56.0%	531	53.0%
**Domestic travel history**					
Yes	47	29	61.7%	27	57.4%
No	974	540	55.4%	510	52.4%
**International travel history**					
Yes	30	25	83.3%	22	73.3%
No	991	544	54.9%	515	51.9%
**Bednet use**					
Yes	648	347	53.5%	334	51.5%
No	373	222	59.5%	203	54.4%

**Fig 5:**
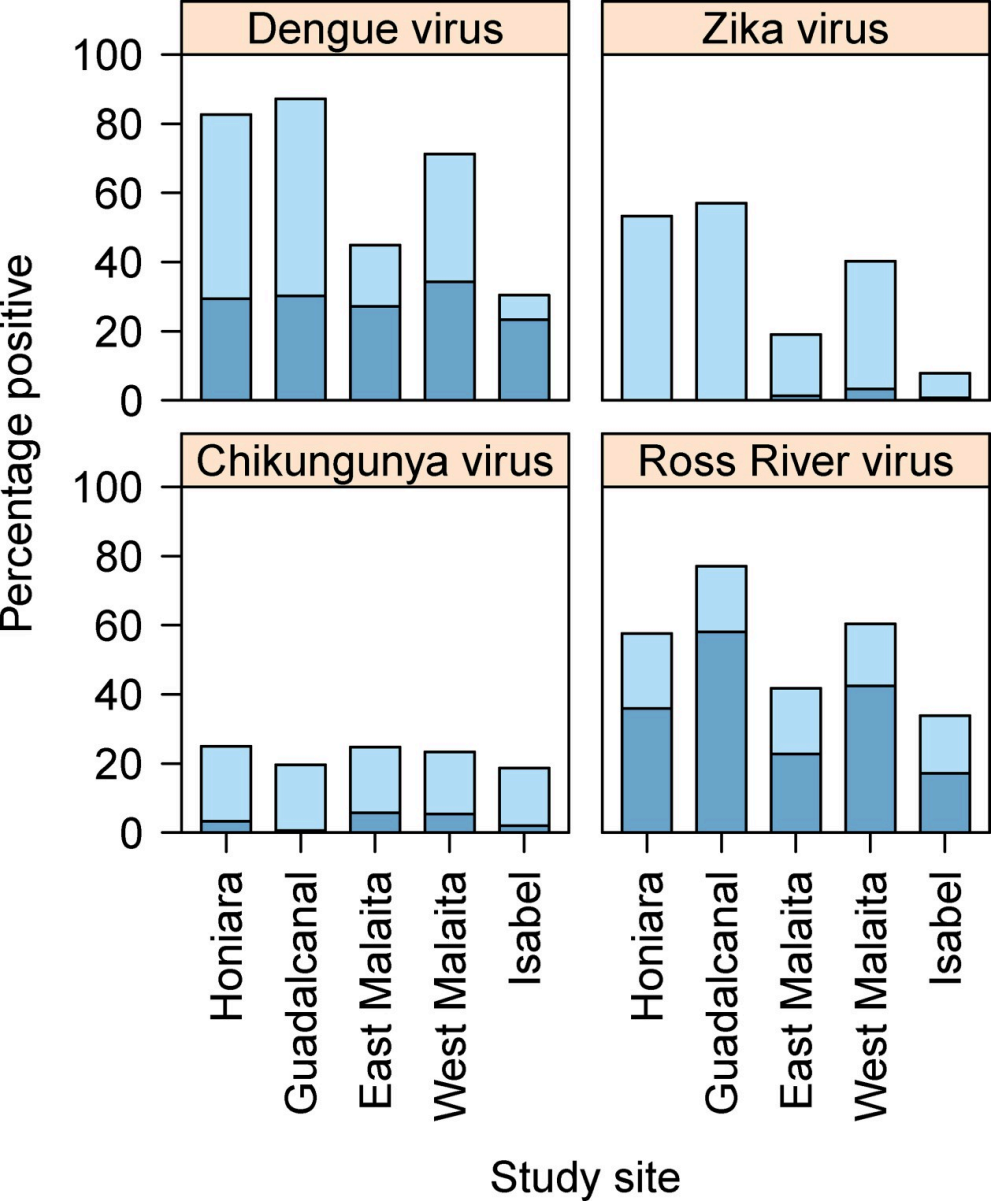
The seroprevalence of flaviviruses and alphavirus across different study sites in the Solomon Islands. The darker blue colour represents samples that were positive for that particular virus alone. The lighter blue colour represents samples that were positive for both of the flaviviruses (dengue and Zika) or both of the alphaviruses (chikungunya and Ross River).

Seropositivity varied across study sites for all arboviruses tested (Fig 5). The prevalence of antibody positive individuals to one or more of the flaviviruses (either DENV or ZIKV) and the alphaviruses (CHIKV or RRV) was highest in the villages of Guadalcanal, Honiara and West Malaita (Figs 2 and 3). In East Malaita and Isabel, the prevalence of antibodies recognising flaviviruses and alphaviruses was relatively lower than the other sites. The prevalence of antibody positive individuals increased by age categories, peaking in the 21–30 years group for DENV and 31–40 years for ZIKV and RRV (Fig 4). Within the flaviviruses, there was significantly more residents that were solely seropositive for DENV compared with ZIKV (χ^2^ = 309.71, df = 1, p <0.0001). Within the alphaviruses, there was significantly more residents that were solely seropositive for RRV compared with CHIKV (χ^2^ = 300.55, df = 1, p <0.0001).

## Discussion

This is the first study to systematically test for DENV, ZIKV, CHIKV and RRV in multiple urban and rural sites across the Solomon Islands. These are important results given the transmission and geographic distributions of DENV, ZIKV, CHIKV and RRV have not historically been well documented in Solomon Islands.

This study had some limitations. Cross-reactivity of IgG antibodies amongst flaviviruses and alphaviruses is well documented and is a confounding factor for serological studies investigating the seroprevalence of arboviruses. All samples that were positive for both DENV/ZIKV or CHIKV/RRV were recorded as flavivirus or alphavirus positive, respectively. Further testing using methods such as the plaque reduction and neutralization test (PRNT) were not used, as it was not feasible to test the large number of participants included in the study. However, seropositivity to all four arboviruses were detected in the absence of antibodies to the other flavi- or alphavirus included in the analysis. Thus, circulation of all four of the targeted arboviruses was confirmed. Serology based on IgG antibodies indicates that the resident had previously been infected with the arbovirus. IgG antibodies can be detected for years or possibly even are lifelong [49], thus the current study cannot indicate when people were infected.

As such, we consider the serological results for the flaviviruses (DENV and ZIKV) alongside an understanding of prior epidemiological data. The first reported DENV outbreak was in 1982 in Honiara [50]. After which DENV local transmission was not reported within Solomon Islands for almost 2 decades. In the early 2000’s 3 cases of DENV were detected in travellers returning from the Solomon Islands (one was infected with DENV-2 in 2001 [51], one infected with DENV-4 in 2007 [52] and another with DENV-1 in 2008 [53]). In 2013, there was a DENV-3 outbreak with 5,254 suspected cases [12,15,54]. In 2016–17, there was a DENV-2 and DENV-3 outbreak with 12,329 suspected cases [16,17]. In April 2016, the DENV seroprevalence by IgG ELISA was 88% in 78 Honiara residents [17]. Limited testing for ZIKV exposure has been conducted in the Solomon Islands. Zika transmission was initially reported in early 2015 [55], with continued cases reported throughout the year [56,57], and transmission likely into 2016, when ZIKV was detected in a traveller that had returned from the Solomon Islands [27]. Sequentially, Darcy et al. [17] detected a seroprevalence of 7% during 2016.

This study confirms a high seroprevalence to flaviviruses in the Solomon Islands (56% overall). The site of Honiara allowed a direct comparison with the previous seroprevalence survey [17]. Here, residents were 29% solely seropositive for DENV, 0% solely seropositive for ZIKV and 49% were seropositive for DENV/ZIKV. Considering that there has been limited evidence from the syndromic surveillance system indicating epidemic circulation of ZIKV in Honiara, this suggests that the cross-reacting antibodies are most likely DENV infections. This is also supported by the similar (being 88%) seroprevalence of DENV antibodies observed in 2016 [17].

Regarding the alphaviruses (CHIKV and RRV), there has been limited testing and little epidemiological data is available. An initial case of CHIKV was detected during the 2013 DENV outbreak [22]. Subsequentially, Darcy et al. [17] reported a seroprevalence in Honiara of 0.9% in 2016. Evidence of RRV transmission in the Solomon Islands dates back to 1975 [18], yet subsequently there have been no reports of RRV transmission.

This study found a high seroprevalence of alphaviruses in the Solomon Islands (53% overall). Continuing with the example of Honiara, residents were 3% solely seropositive for CHIKV, 36% solely seropositive for RRV and 22% seropositive for cross-reacted RRV/CHIKV. The cross-reacting samples should be interpreted with caution. Regarding CHIKV, the low percentage of sole seropositives indicates limited prior transmission. It is likely that CHIKV only circulated punctually like in most other Pacific islands. More residents were solely seropositive to RRV antibodies, which supports the hypothesis that RRV transmission is endemic, rather than epidemic. It is likely that RRV is circulating throughout the Solomon Islands being possibly undiagnosed or misdiagnosed as DENV due to the overlap in symptoms [38].

The seroprevalence of both flavi- and alphaviruses was significantly influenced by village and age, and are discussed in relation to the two most common arboviruses in the Solomon Islands (being DENV and RRV). There are likely a confluence of factors present in each village that influence the amount of potential transmission including the vectors present, density of the human population, prior exposure of the population to arboviruses and the influx of travellers. Dengue seroprevalence was highest from the more heavily populated villages in Honiara, Guadalcanal and West Malaita. This is not surprising considering the domesticated nature of the primary vector, *Ae*. *aegypti*. Other vectors in the Solomon Islands include *Ae*. *albopictus* and *Ae*. *hebrideus*, a vector in the outer islands of Rennell, Bellona, Ontong Java, Sikaiana and Temotu [58]. *Aedes albopictus* is found in all provinces in which the survey took place, with *Ae*. *aegypti* found in all provinces except Isabel. The high seroprevalence of DENV documented in this study suggests that despite reactive vector control in the Solomon Islands, herd immunity may still be a driver of DENV outbreak dynamics in the Solomon Islands [17]. A large outbreak of DENV had occurred only 3 years prior to the sample collection, and as such people across all age groups were seropositive indicating that transmission had been recent.

Regarding RRV, extremely little is known about the transmission of this arbovirus in the Pacific. Here, relatively higher seroprevalences were recorded in Guadalcanal and West Malaita, suggesting possibly a greater density of enzootic hosts, outside the capital city. The potential enzootic reservoirs in the Solomon Islands are likely to be pigs, rats and birds [30,40], noting that pigs are one of the more common animals kept by residents [59]. The suggestion of ongoing endemic circulation of RRV is supported by the seropositive age profile, for which there is a progressive increase in seroprevalence with age.

The transmission of DENV, ZIKV and CHIKV occur sporadically in the Pacific, and usually an infected traveller attributed as responsible for introducing the pathogen, as such mobility of the study population is of interest. Improvements in airline travel links in, out and across the Pacific will make it easier to introduce new viruses in the region, as airline travel has been associated with the introduction of virus into receptive areas [60]. Phylogenetic sequences of DENV infections provide evidence that serotypes of DENV are re-introduced into the Pacific region often from Southeast Asia, and then move from country-to-country within the region by infected travellers [61]. Here, travel data collected was a proxy to indicate the strength of travel by residents within each region. Travel was strongly correlated with village but was excluded from the final model due to multicollinearity. This data was difficult to relate directly to seropositivity. Of note in this study, residents from East Malaita had the highest proportion of international travel (7%), and this was mostly concentrated in the Atoifi Campus village where 13% of residents had previously travelled internationally. The Atoifi Campus village hosts the Atoifi Hospital and is also the location of Atoifi College of Nursing. Staff from the hospital and College of Nursing were included as participants in this study. The staff travel internationally for ongoing education and professional reasons. Therefore, although this study site is remote, it does demonstrated that across Solomon Islands and indeed the Pacific, there may be education, health or other ‘hubs’ in seemingly remote locations that facilitate atypical levels of international travel that need to be considered in relation to disease transmission and distribution.

In the Solomon Islands, records of arbovirus occurrence and outbreaks are often incomplete, and confounded by the lack of accurate diagnostics for testing. This means that reported case numbers are often based on clinical presentation. This lack of complete and timely information about arbovirus transmission and may result in postponed, or even no response measures taken. This leads to greater risk of transmission and impact. The large DENV outbreaks experienced in 2013 and 2016–17 did overwhelm the country’s fragile health system [62].

### Conclusion

The most prevalent arboviruses in the Solomon Islands were DENV and RRV. The high seroprevalence of DENV confirms that high levels of immunity of the population was reached during the recent outbreaks. Regarding RRV, this is the first survey to document how extensive RRV transmission is throughout the country. It is likely that undetected RRV transmission was ongoing. There is a real need to increase the diagnostic capacities for each of these arboviruses to support effective case management and to provide timely information to inform vector control efforts. The Solomon Islands remains vulnerable to outbreaks of DENV, ZIKV and CHIKV, with endemic transmission of RRV.

## Supporting information

S1 Text Visualization of domestic and international travel reported by participants in the Solomon Islands epidemiological survey. **Fig A: Domestic travel reported by participants in the Solomon Islands epidemiological survey.** Circles represent locations of participants and circle size is proportional to the number of participants with domestic travel history in the two weeks preceding the survey. One-way or returning arrows represent inter- and intra-Provincial travel, respectively, with the width of the arrow proportional to the number of people that moved between two locations. The base map was obtained from http://diva-gis.org/data. **Fig B: International travel reported by participants in the Solomon Islands epidemiological survey.** Return travel from the Solomon Islands to other countries are represented by an arrow to the destination country, with the arrow width proportional to the number of people that travelled between countries. The base map was obtained from http://diva-gis.org/data.
(DOCX)

## References

[1] Franklinos LHV, Jones KE, Redding DW, Abubakar I. The effect of global change on mosquito-borne disease. Lancet Infect Dis. 2019;19(9):e302–e12. http://www.sciencedirect.com/science/article/pii/S1473309919301616 doi: 10.1016/S1473-3099(19)30161-6 31227327

[2] Brady OJ, Hay SI. The global expansion of dengue: How *Aedes aegypti* mosquitoes enabled the first pandemic arbovirus. Annu Rev Entomol. 2020;65(1):191–208. https://www.annualreviews.org/doi/abs/10.1146/annurev-ento-011019-024918 31594415 10.1146/annurev-ento-011019-024918

[3] Bhatt S, Gething PW, Brady OJ, Messina JP, Farlow AW, Moyes CL, et al. The global distribution and burden of dengue. Nature. 2013;496(7446):504–7. doi: 10.1038/nature12060 23563266 PMC3651993

[4] Lambin EF, Tran A, Vanwambeke SO, Linard C, Soti V. Pathogenic landscapes: Interactions between land, people, disease vectors, and their animal hosts. Int J Health Geogr. 2010;9(1):54. 10.1186/1476-072X-9-54 20979609 PMC2984574

[5] Mayer SV, Tesh RB, Vasilakis N. The emergence of arthropod-borne viral diseases: A global prospective on dengue, chikungunya and zika fevers. Acta Trop. 2017;166:155–63. http://www.sciencedirect.com/science/article/pii/S0001706X16306246 doi: 10.1016/j.actatropica.2016.11.020 27876643 PMC5203945

[6] Roth A, Mercier A, Lepers C, Hoy D, Duituturaga S, Benyon E, et al. Concurrent outbreaks of dengue, chikungunya and Zika virus infections–an unprecedented epidemic wave of mosquito-borne viruses in the Pacific 2012–2014. Eurosurveillance. 2014;19(41):20929. https://www.eurosurveillance.org/content/10.2807/1560-7917.ES2014.19.41.20929 25345518 10.2807/1560-7917.es2014.19.41.20929

[7] Paixão ES, Teixeira MG, Rodrigues LC. Zika, chikungunya and dengue: the causes and threats of new and re-emerging arboviral diseases. BMJ Global Health. 2018;3(Suppl 1):e000530. http://gh.bmj.com/content/3/Suppl_1/e000530.abstract doi: 10.1136/bmjgh-2017-000530 29435366 PMC5759716

[8] Russell RC. Ross river virus: ecology and distribution. Annu Rev Entomol. 2002;47:1–31. doi: 10.1146/annurev.ento.47.091201.145100 11729067

[9] Kraemer MUG, Reiner RC, Brady OJ, Messina JP, Gilbert M, Pigott DM, et al. Past and future spread of the arbovirus vectors *Aedes aegypti* and *Aedes albopictus*. Nature Microbiology. 2019;4(5):854–63. 10.1038/s41564-019-0376-y 30833735 PMC6522366

[10] Schoenig E. Distribution of three species of *Aedes* (Stegomyia) carriers of virus diseases on the main island of Papua New Guinea. The Philippine Scientist. 1972;9:61–82.

[11] Elliott SA. *Aedes albopictus* in the Solomon and Santa Cruz Islands, South Pacific. Trans R Soc Trop Med Hyg. 1980;74(6):747–8. http://trstmh.oxfordjournals.org/content/74/6/747.abstract doi: 10.1016/0035-9203(80)90192-3 7210128

[12] Shortus M, Musto J, Bugoro H, Butafa C, Aio A, Joshua C. Vector-control response in a post-flood disaster setting, Honiara, Solomon Islands, 2014. Western Pacific Surveillance and Response Journal. 2016;7(1):1–6. http://ojs.wpro.who.int/ojs/index.php/wpsar/article/view/390 doi: 10.5365/WPSAR.2015.6.2.010 27757255 PMC5052898

[13] Demok S, Endersby-Harshman N, Vinit R, Timinao L, Robinson LJ, Susapu M, et al. Insecticide resistance status of *Aedes aegypti* and *Aedes albopictus* mosquitoes in Papua New Guinea. Parasit Vectors. 2019;12(1):333. doi: 10.1186/s13071-019-3585-6 31269965 PMC6609403

[14] Ryan PA, Do KA, Kay BH. Definition of Ross River virus vectors at Maroochy Shire, Australia. J Med Entomol. 2000;37(1):146–52. doi: 10.1603/0022-2585-37.1.146 15218919

[15] Nogareda F, Joshua C, Sio A, Shortus M, Dalipanda T, Durski K, et al. Ongoing outbreak of dengue serotype-3 in Solomon Islands, January to May 2013. Western Pacific Surveillance and Response Journal. 2013;4(3):1–5. http://ojs.wpro.who.int/ojs/index.php/wpsar/article/view/206 doi: 10.5365/WPSAR.2013.4.3.004 24319611 PMC3853998

[16] Craig AT, Joshua CA, Sio AR, Teobasi B, Dofai A, Dalipanda T, et al. Enhanced surveillance during a public health emergency in a resource-limited setting: Experience from a large dengue outbreak in Solomon Islands, 2016–17. PLoS ONE. 2018;13(6):e0198487. 10.1371/journal.pone.0198487 29879179 PMC5991673

[17] Darcy AW, Kanda S, Dalipanda T, Joshua C, Shimono T, Lamaningao P, et al. Multiple arboviral infections during a DENV-2 outbreak in Solomon Islands. Trop Med Health. 2020;48(1):33. 10.1186/s41182-020-00217-8 32435149 PMC7225641

[18] Tesh RB, Gajdusek DC, Garruto RM, Cross JH, Rosen L. The distribution and prevalence of group a arbovirus neutralizing antibodies among human populations in Southeast Asia and the Pacific Islands. Am J Trop Med Hyg. 1975;24(4):664–75. http://www.ajtmh.org/content/journals/10.4269/ajtmh.1975.24.664 1155702 10.4269/ajtmh.1975.24.664

[19] Horwood P, Bande G, Dagina R, Guillaumot L, Aaskov J, Pavlin B. The threat of chikungunya in Oceania. Western Pacific surveillance and response journal: WPSAR. 2013;4(2):8. doi: 10.5365/WPSAR.2013.4.2.003 24015365 PMC3762969

[20] Dupont-Rouzeyrol M, Caro V, Guillaumot L, Vazeille M, D’Ortenzio E, Thiberge J-M, et al. Chikungunya virus and the mosquito vector *Aedes aegypti* in New Caledonia (South Pacific Region). Vector-Borne and Zoonotic Diseases. 2012;12(12):1036–41. https://www.liebertpub.com/doi/abs/10.1089/vbz.2011.0937 23167500 10.1089/vbz.2011.0937

[21] Aubry M, Teissier A, Roche C, Richard V, Yan AS, Zisou K, et al. Chikungunya outbreak, French Polynesia, 2014. Emerg Infect Dis. 2015;21(4):724. doi: 10.3201/eid2104.141741 25811534 PMC4378499

[22] Mangum BP, Mangum T, Mangum AP. A case report of chikungunya versus dengue during an acute outbreak of dengue fever in the Solomon Islands, 2013. Archives of Immunology and Allergy. 2018;1(1):41–5.

[23] Duffy MR, Chen T-H, Hancock WT, Powers AM, Kool JL, Lanciotti RS, et al. Zika virus outbreak on Yap Island, Federated States of Micronesia. N Engl J Med. 2009;360(24):2536–43. https://www.nejm.org/doi/full/10.1056/NEJMoa0805715 19516034 10.1056/NEJMoa0805715

[24] Cao-Lormeau V-M, Blake A, Mons S, Lastère S, Roche C, Vanhomwegen J, et al. Guillain-Barré syndrome outbreak associated with Zika virus infection in French Polynesia: a case-control study. Lancet. 2016;387(10027):1531–9. http://www.sciencedirect.com/science/article/pii/S0140673616005626 doi: 10.1016/S0140-6736(16)00562-6 26948433 PMC5444521

[25] Cao-Lormeau V-M, Roche C, Teissier A, Robin E, Berry A-L, Mallet H-P, et al. Zika virus, French Polynesia, South Pacific, 2013. Emerg Infect Dis. 2014;20(6):1085–6. https://pubmed.ncbi.nlm.nih.gov/24856001 doi: 10.3201/eid2006.140138 24856001 PMC4036769

[26] Musso D, Nilles EJ, Cao-Lormeau VM. Rapid spread of emerging Zika virus in the Pacific area. Clinical Microbiology and Infection. 2014;20(10):O595–O6. 10.1111/1469-0691.12707 24909208

[27] Rafiei N, Hajkowicz K, Redmond A, Taylor C. First report of Zika virus infection in a returned traveller from the Solomon Islands. Med J Aust. 2016;204(5):186–. https://onlinelibrary.wiley.com/doi/abs/10.5694/mja15.01275 26985845 10.5694/mja15.01275

[28] Baud D, Gubler DJ, Schaub B, Lanteri MC, Musso D. An update on Zika virus infection. Lancet. 2017;390(10107):2099–109. http://www.sciencedirect.com/science/article/pii/S0140673617314502 doi: 10.1016/S0140-6736(17)31450-2 28647173

[29] Harley D, Ritchie S, Bain C, Sleigh A. Risks for Ross River virus disease in tropical Australia. Int J Epidemiol. 2005;34:548–55. doi: 10.1093/ije/dyh411 15659466

[30] Kain MP, Skinner EB, van den Hurk AF, McCallum H, Mordecai EA. Physiology and ecology combine to determine host and vector importance for Ross River virus. eLife. 2021;10:e67018. 10.7554/eLife.67018 34414887 PMC8457839

[31] Stephenson EB, Peel AJ, Reid SA, Jansen CC, McCallum H. The non-human reservoirs of Ross River virus: a systematic review of the evidence. Parasit Vectors. 2018;11(1):188. 10.1186/s13071-018-2733-8 29554936 PMC5859426

[32] Fauran P, Donaldson M, Harper J, Oseni RA, Aaskov JG. Characterization of Ross River viruses isolated from patients with polyarthritis in New Caledonia and Wallis and Futuna Islands. Am J Trop Med Hyg. 1984;33(6):1228–31. http://www.ajtmh.org/content/journals/10.4269/ajtmh.1984.33.1228 6095694 10.4269/ajtmh.1984.33.1228

[33] Aaskov JG, Mataika JU, Lawrence GW, Rabukawaqa V, Tucker MM, Miles JAR, et al. An epidemic of Ross River virus infection in Fiji, 1979. Am J Trop Med Hyg. 1981;30(5):1053–9. https://www.ajtmh.org/content/journals/10.4269/ajtmh.1981.30.1053 7283004 10.4269/ajtmh.1981.30.1053

[34] Rosen L, Gubler DJ, Bennett PH. Epidemic polyarthritis (Ross River) virus infection in the Cook Islands. Am J Trop Med Hyg. 1981;30(6):1294–302. https://www.ajtmh.org/content/journals/10.4269/ajtmh.1981.30.1294 7325286 10.4269/ajtmh.1981.30.1294

[35] Tesh RB, McLean RG, Shroyer DA, Calisher CH, Rosen L. Ross River virus (Togaviridae: Alphavirus) infection (epidemic polyarthritis) in American Samoa. Trans R Soc Trop Med Hyg. 1981;75(3):426–31. http://www.sciencedirect.com/science/article/pii/0035920381901127 doi: 10.1016/0035-9203(81)90112-7 7324110

[36] Klapsing P, MacLean JD, Glaze S, McClean KL, Drebot MA, Lanciotti RS, et al. Ross River virus disease reemergence, Fiji, 2003–2004. Emerging Infectious Deseases. 2005;11(4):613–5. doi: 10.3201/eid1104.041070 15829203 PMC3320333

[37] Lau C, Weinstein P, Slaney D. Imported cases of Ross River virus disease in New Zealand–A travel medicine perspective. Travel Med Infect Dis. 2012;10(3):129–34. http://www.sciencedirect.com/science/article/pii/S1477893912000634 doi: 10.1016/j.tmaid.2012.04.001 22579017

[38] Lau C, Aubry M, Musso D, Teissier A, Paulous S, Desprès P, et al. New evidence for endemic circulation of Ross River virus in the Pacific Islands and the potential for emergence. International Journal of Infectious Diseases. 2017;57:73–6. http://www.sciencedirect.com/science/article/pii/S1201971217300449 doi: 10.1016/j.ijid.2017.01.041 28188934

[39] Aubry M, Finke J, Teissier A, Roche C, Broult J, Paulous S, et al. Silent circulation of Ross River virus in French Polynesia. International Journal of Infectious Diseases. 2015;37:19–24. http://www.sciencedirect.com/science/article/pii/S1201971215001393 doi: 10.1016/j.ijid.2015.06.005 26086687

[40] Togami E, Gyawali N, Ong O, Kama M, Cao-Lormeau V-M, Aubry M, et al. First evidence of concurrent enzootic and endemic transmission of Ross River virus in the absence of marsupial reservoirs in Fiji. International Journal of Infectious Diseases. 2020;96:94–6. doi: 10.1016/j.ijid.2020.02.048 32114197

[41] Wilson AL, Courtenay O, Kelly-Hope LA, Scott TW, Takken W, Torr SJ, et al. The importance of vector control for the control and elimination of vector-borne diseases. PLoS Negl Trop Dis. 2020;14(1):e0007831. doi: 10.1371/journal.pntd.0007831 31945061 PMC6964823

[42] WHO. Malaria surveillance, monitoring & evaluation: a reference manual. Geneva: World Health Organization, 2018. https://apps.who.int/iris/handle/10665/272284

[43] Russell TL, Grignard L, Apairamo A, Kama N, Bobogare A, Drakeley C, et al. Getting to zero: micro-foci of malaria in the Solomon Islands requires stratified control. Malar J. 2021;20(1):248. doi: 10.1186/s12936-021-03779-y 34090430 PMC8180101

[44] Burnham KP, Anderson DR. Multimodel inference: understanding AIC and BIC in model selection. Soc Methods Res. 2004;33(2):261–304.

[45] Burnham KP, Anderson DR. Model selection and inference: a practical information-theoretic approach. 2nd Edn. New York, USA: Springer-Verlag; 2002.

[46] Link WA, Barker RJ. Model weights and the foundations of multimodel inference. Ecology. 2006;87(10):2626–35. doi: 10.1890/0012-9658(2006)87[2626:mwatfo]2.0.co;2 17089670

[47] Pinheiro JC, Bates DH. Mixed-effects models in S and S-PLUS. New York: Springer; 2000.

[48] Russell TL, Lwetoijera DW, Knols BGJ, Takken W, Killeen GF, Ferguson HM. Linking individual phenotype to density-dependent population growth: the influence of body size on the population dynamics of malaria vectors. Proc Biol Sci. 2011;278:3142–51. http://rspb.royalsocietypublishing.org/content/early/2011/03/05/rspb.2011.0153.abstract 21389034 10.1098/rspb.2011.0153 PMC3158942

[49] Kerkhof K, Falconi-Agapito F, Van Esbroeck M, Talledo M, Ariën KK. Reliable serological diagnostic tests for arboviruses: feasible or utopia? Trends in Microbiology. 2020;28(4):276–92. https://www.sciencedirect.com/science/article/pii/S0966842X19302914 doi: 10.1016/j.tim.2019.11.005 31864844

[50] Darcy AW, Clothier H, Phillips D, Bakote’e B, Stewart T. Solomon Islands dengue seroprevalence study—previous circulation of dengue confirmed. Papua and New Guinea Medical Journal. 2001;44(1–2):43–7. 12418677

[51] Oishi K, Saito M, Mapua CA, Natividad FF. Dengue illness: clinical features and pathogenesis. Journal of Infection and Chemotherapy. 2007;13(3):125–33. https://www.sciencedirect.com/science/article/pii/S1341321X07708565 doi: 10.1007/s10156-007-0516-9 17593497

[52] Shu PY, Su CL, Liao TL, Yang CF, Chang SF, Lin CC, et al. Molecular characterization of dengue viruses imported into Taiwan during 2003–2007: geographic distribution and genotype shift. Am J Trop Med Hyg. 2009;80(6):1039–46. https://www.ajtmh.org/view/journals/tpmd/80/6/article-p1039.xml?rskey=ohD5mH&result=1 19478273

[53] Warrilow D, Northill JA, Pyke AT. Sources of dengue viruses imported into Queensland, australia, 2002–2010. Emerg Infect Dis. 2012;18(11):1850–7. https://dx.doi.org/10.3201%2Feid1811.120014 23092682 10.3201/eid1811.120014 PMC3559152

[54] Cao-Lormeau V-M, Roche C, Musso D, Mallet H-P, Dalipanda T, Dofai A, et al. Dengue virus type 3, South Pacific Islands, 2013. Emerg Infect Dis J. 2014;20(6):1034. https://wwwnc.cdc.gov/eid/article/20/6/13-1413_article 10.3201/eid2006.131413 PMC4036764 24856252

[55] Craig AT, Butler MT, Pastore R, Paterson BJ, Durrheim DN. Acute flaccid paralysis incidence and Zika virus surveillance, Pacific Islands. Bull World Health Organ. 2017;95(1):69–75. https://pubmed.ncbi.nlm.nih.gov/28053366 doi: 10.2471/BLT.16.171892 28053366 PMC5180343

[56] Paixão ES, Barreto F, Teixeira MdG, Costa MdCN, Rodrigues LC. History, epidemiology, and clinical manifestations of Zika: A systematic review. Am J Public Health. 2016;106(4):606–12. https://ajph.aphapublications.org/doi/abs/10.2105/AJPH.2016.303112 26959260 10.2105/AJPH.2016.303112 PMC4816002

[57] Musso D, Gubler Duane J. Zika virus. Clin Microbiol Rev. 2016;29(3):487–524. doi: 10.1128/CMR.00072-15 27029595 PMC4861986

[58] Belkin JN. The mosquitoes of the South Pacific (Diptera, Culicidae). Berkeley and Los Angeles: University of California Press; 1962.

[59] Russell TL, Beebe NW, Bugoro H, Apairamo A, Cooper RD, Collins FH, et al. Determinants of host feeding success by *Anopheles farauti*. Malar J. 2016;15(1):1–9. 10.1186/s12936-016-1168-y 26964528 PMC4785651

[60] Tian H, Sun Z, Faria NR, Yang J, Cazelles B, Huang S, et al. Increasing airline travel may facilitate co-circulation of multiple dengue virus serotypes in Asia. PLoS Negl Trop Dis. 2017;11(8):e0005694. doi: 10.1371/journal.pntd.0005694 28771468 PMC5542384

[61] Inizan C, O’Connor O, Worwor G, Cabemaiwai T, Grignon J-C, Girault D, et al. Molecular characterization of dengue type 2 outbreak in Pacific Islands Countries and Territories, 2017–2020. Viruses. 2020;12(10):1081. https://www.mdpi.com/1999-4915/12/10/1081 doi: 10.3390/v12101081 32992973 PMC7601490

[62] Goulolo ND, Bugoro H, Whittaker M, Larkins S, Harrington H, Carlisle K, et al. Perspectives of nurses about factors affecting quality of care at the Solomon Islands National Referral Hospital during the 2016–2017 dengue outbreak: a qualitative study. Asia Pacific Journal of Public Health. 2021:10105395211036266. 10.1177/10105395211036266 34334032

