# Supplementary material for: Seroprevalence of dengue, Zika, chikungunya and Ross River viruses across the Solomon Islands
Source: PLoS Negl Trop Dis. 2022 Feb 10;16(2):e0009848. doi: 10.1371/journal.pntd.0009848 (PMC8865700; doi:10.1371/journal.pntd.0009848)

***S1 Text* is a supplemental file for:** Russell TL, Horwood PF, Harrington H, Apairamo A, Kama N, Bobogare A, MacLaren D, Burkot TR. **Seroprevalence of dengue, Zika, chikungunya and Ross River viruses across the Solomon Islands.** PLoS Neglected Tropical Diseases. 2022.

The demographic survey captured information on domestic travel in the past 14 days and international travel at any time. Travel history was strongly correlated with village and the patterns are visualized here.

**Fig A. Domestic travel reported by participants in the Solomon Islands epidemiological survey.** Circles represent locations of participants and circle size is proportional to the number of participants with domestic travel history in the two weeks preceding the survey. One-way or returning arrows represent inter- and intra-Provincial travel, respectively, with the width of the arrow proportional to the number of people that moved between two locations. The base map was obtained from <http://diva-gis.org/data>.


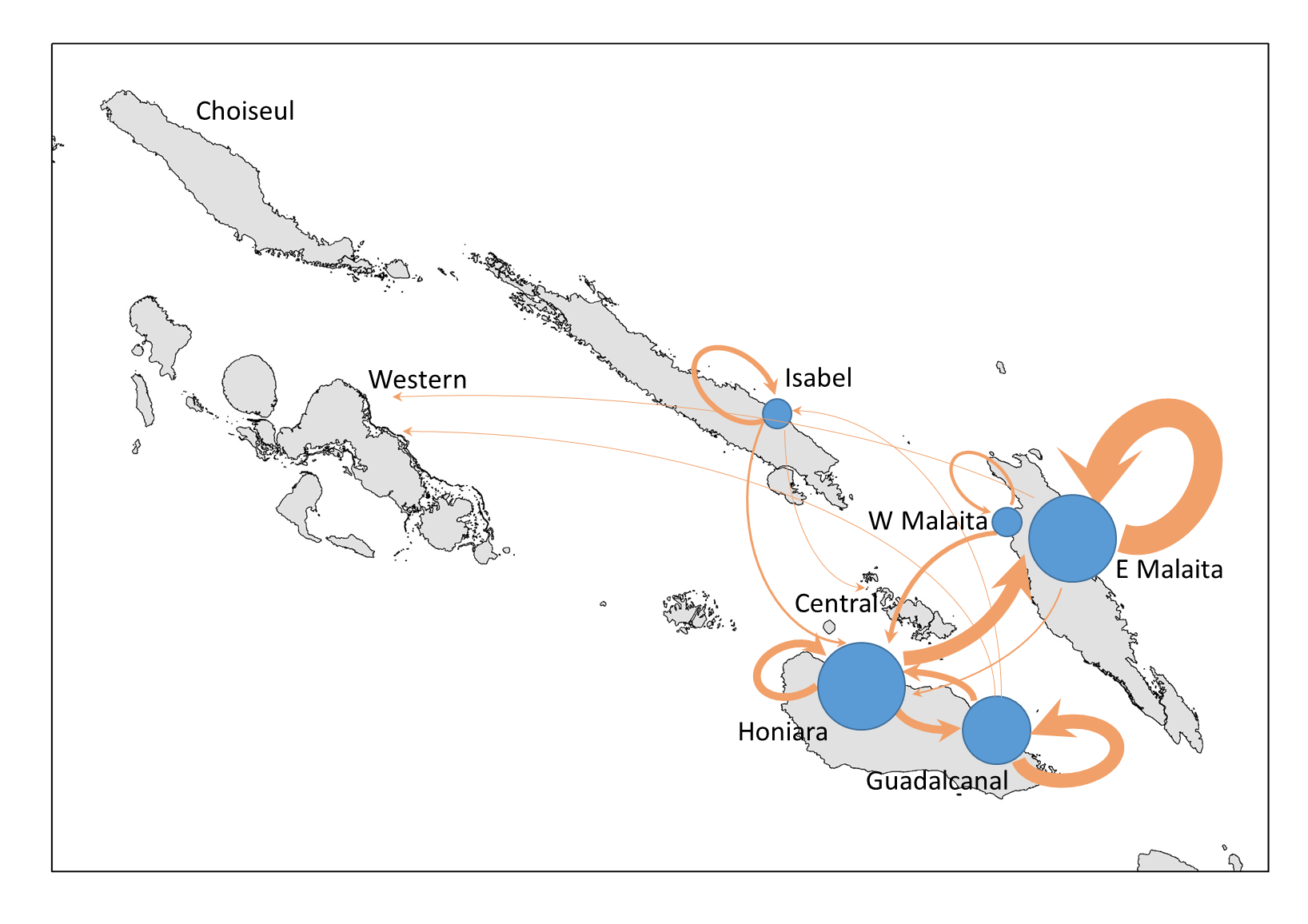


**Fig B. International travel reported by participants in the Solomon Islands epidemiological survey.** Return travel from the Solomon Islands to other countries are represented by an arrow to the destination country, with the arrow width proportional to the number of people that travelled between countries. The base map was obtained from <http://diva-gis.org/data>.


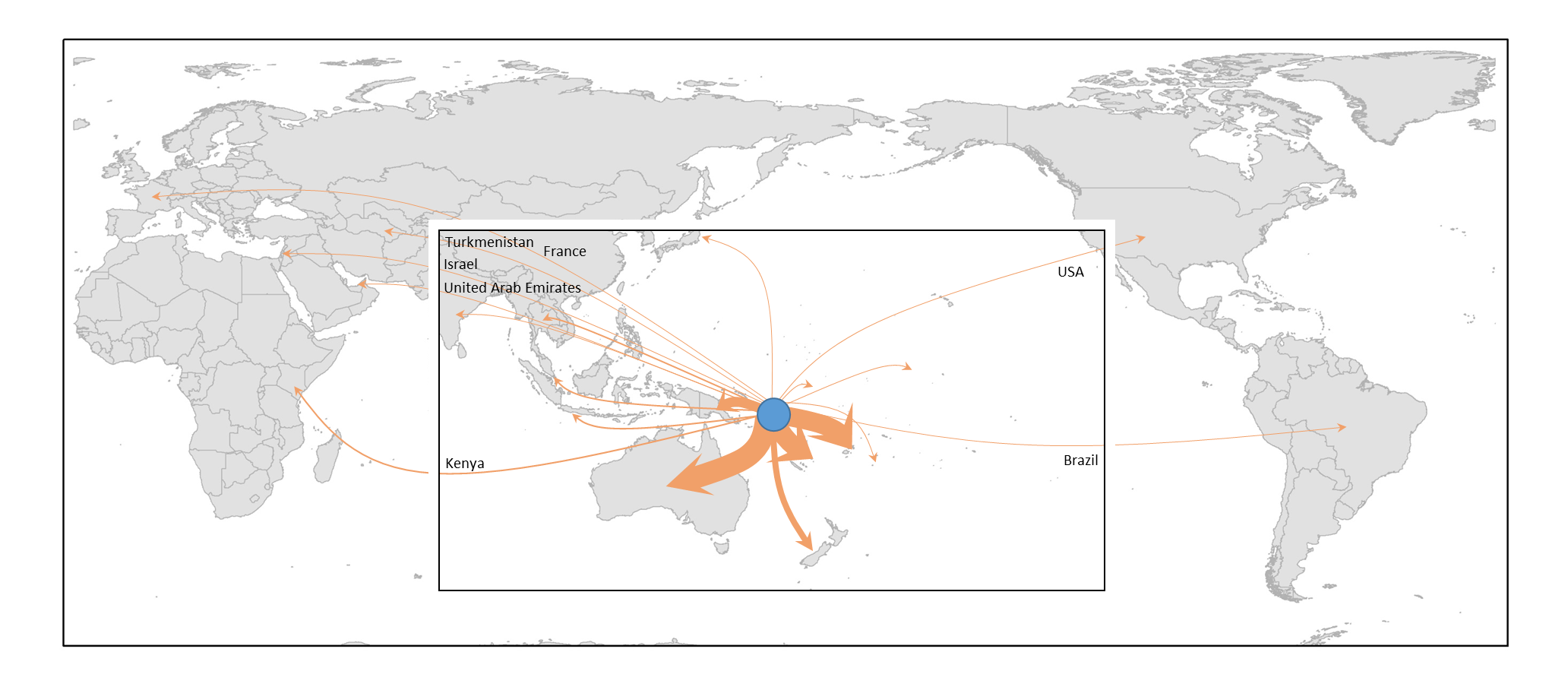

Supplement: S1 Text — Fig A: Domestic travel reported by participants in the Solomon Islands epidemiological survey. Circles represent locations of participants and circle size is proportional to the number of participants with domestic travel history in the two weeks preceding the survey. One-way or returning arrows represent inter- and intra-Provincial travel, respectively, with the width of the arrow proportional to the number of people that moved between two locations. The base map was obtained from http://diva-gis.org/data. Fig B: International travel reported by participants in the Solomon Islands epidemiological survey. Return travel from the Solomon Islands to other countries are represented by an arrow to the destination country, with the arrow width proportional to the number of people that travelled between countries. The base map was obtained from http://diva-gis.org/data. (DOCX) [file pntd.0009848.s001.docx]
